# Medical News and Miscellany

**Published:** 1893-06

**Authors:** 


### Medical News and Miscellany.

Removals.—Dr. W. T. Jones has removed from Hockheim to San Marcos, Texas.

---

Dr. J. E. Roberts, at Lone Star, Texas, lost his life in a fire which occurred on the 23d May ult.

---

Dr. W. S. Evans, of Jewett, Texas, has returned from taking a post-graduate course at the New Orleans Polyclinic.

---

Dr. E. M. Johnson, late Dean and Professor of Obstetrics, Kansas City Medical College, died in Kansas City in April.

**Married** in Lampasas May —, ult., Dr. J. H. McCaleb, of Webberville, to Miss Mattie Hamilton, a sister of Dr. Hamilton, of Lampasas.

---

**The Medical Mirror** is running an advertisement for a remedy warranted to cure "that tired feeling." American medical editors, "where are we at?"—*St. Joseph Medical Herald.*

---

**A Thoroughly Competent Druggist**, twenty-eight years of age, married, desires a situation. Speaks Spanish and English, and has had ten years experience. Best testimonials as to character, and habits and qualifications. Address "D," care this journal, Austin, Texas.

---

**The** Ohio State Board of Health insists that kissing shall be suppressed, "because it conveys the bacilli of disease." Now we know what causes that peculiar sensation when the ruby lips of Rebecca Merlindy Johnson are rooting around under our white-horse moustache. It is the disease bacilli scampering down our back. Dont let it happen again, Merlindy.—*Ex.*

---

**For Sale.**—Dr. W. B. Anderson, whose card appeared in the JOURNAL last winter, now, since his expected trip is close at hand, offers his property for less than cost. This is certainly an unusual opportunity, for the Doctor can put a physician into an annual $2000 practice at once without opposition. Address him at CONTENT, RUNNELS CO., TEXAS.

---

**Prof. H. A. West, M. D.**, Secretary of the Texas State Medical Association, requests all members who participated in the discussion of papers at the Galveston meeting, to send him, at once, at outline of their remarks, brief and to the point. Also requests members who are in possession of any facts in connection with the mortuary record of members which have been omitted, or who are cognizant of any errors in the list of deceased, to correspond with him.

---

**Death of a Veteran Texas Physician.**—THE JOURNAL is pained to chronicle the death of another of the old land marks of pioneer medicine in Texas. Dr. J. E. Walker, of Georgetown, died in that city on 31st of May, ult., of gastro-enteritis, after a protracted illness. Dr. Walker was a brother of Judge A. S. Walker, of Austin, and was one of the oldest and most popular physicians of the State. He had practiced medicine in

Georgetown, and in Williamson county, more than a quarter century. In his death the State sustains a loss which will be widely mourned,— a highly educated and able physician, and an honest man, the noblest work of God.

---

**For Sale.**—One of the best improved and most desirable places in Pontotoc, with a practice worth $2000 a year. House and lot cost $1500 five years ago. As I have made arrangements to go to another point, in order to make a quick sale. I will take $800 for property and practice, one-half down, balance in one and two years. Pontotoc has a fine school, and three good churches. Address:

R. B. ANDERSON,
Pontotoc, Mason county, Texas.

---

**Prof. E. Lanphear, M. D.**, of Kansas City, will deliver a course of lectures in the Chicago Post-Graduate Medical School, this summer, on "Some Achievements in Intra-Cranial Surgery," from the standpoint of his own large experience, larger, perhaps, than that of any American surgeon. In the announcement of this course of lectures, sent out on postal cards, in which course Lawson Tait, Reginald Harrison, Jos. Price, Prof. Lanphear, and others, will take part, the management made the unpardonable mistake of locating Prof. Lanphear at New York, and of omitting the title. of his subject. We make the correction with pleasure.

---

**Bills.**—As usual on sending out the last number of a volume, we will send bills for the next volume, to all our subscribers, giving them the opportunity to *pay in advance*, as so many prefer to do. We also make out and mail *every bill on the books* that is past due, and respectfully ask that, *if convenient*, those in arrears will please remit. We know that money is scarce in the country in summer, and we do not wish our friends to construe this as a *dun*, or think the JOURNAL is disposed to press for payment. Remit if convenient; but do not pay up and stop. It takes money to conduct the JOURNAL, but we are not obliged to inconvenience any one.

---

**No Tongue Depressors Necessary.**—Dr. T. J. Pugh, of Hearne, writes the JOURNAL that in his practice he has no use for a tongue depressor. He seats his patient so as to get a good light on the parts and directs him or her to open the mouth well and at the same time to take a long, deep inspiration. When this is done properly the posterior part of the tongue is as fully depressed as it could be done with any depressor, and there is no "gagging," something that nearly always attends the use of the instrument, and a good view of the fauces and adjacent parts is had. Local applications can be made satisfactorily, also. A little practice will enable any one to well expose the pharynx and fauces voluntarily. Dr. Pugh recommends physicians to give his plan a trial.

---

The Sign Rider.—The Lampasas *Leader* will soon commence the publication of a serial story, entitled "The Sign Rider," which will continue about five months, and will present ranch life in Texas in a form never before undertaken. It is a love-story with a happy moral, and delineates a pursuit, and a life consequent upon it, both of which are fast passing away, and will soon be known only in history, in story, and in song. Were the story without thrilling incidents, hair-breath escapes and blood-curdling horrors, it would not be true to the life on the frontier of Texas.

The story is written by Dr. J. W. Carhart, whom all Texans know. The doctor has fine literary attainments, and is the author of several popular novels. His personal experience in ranch life in Texas will make the "Sign Rider" a splendid story eagerly sought after. The *Leader* is published at Lampasas Springs, Texas. Subscription price, $1.50 a year, in advance.

---

**Prof. J. E. Thompson, M. D.,** Professor of Surgery, Texas Medical College, will spend the summer in England, returning to Galveston September 1st. His address is Eddisbury Hall, Macclesfield, England.

**Prof. Allen J. Smith, M. D.,** Professor Pathology, Texas Medical College, will spend the greater part of the vacation at San Antonio, where his family are at present domiciled,—vibrating between San Antonio and Galveston.

**Prof. Wm. Keiller,** as stated in our last, is in the land o'cakes. His address is Glasgow, 16 Berkeley Terrace, care Mrs. McLaughlin. These gentlemen all being on the staff of the JOURNAL, will contribute, as usual, to its pages.

**Dr. D. Cerna** and Prof. Seth M. Morris, M. D., of the Texas Medical College, also on our staff, will, so far as we know to the contrary, pass the summer in Galveston.

---

Archives of Confederate Medical Service.—Dr. S. H.

Stout, of Cisco, Texas, who held the position of Medical Director of Hospitals of the Confederate Army, has begun, by invitation of the *Editors of the St. Louis Medical and Surgical Journal*, the publication, in that journal, of a series of papers under the head of "Reminiscenses of the Services of Medical Officers of the Confederate States Army, Department of Tennessee," the first paper appearing in the April number.

The editor of DANIEL'S TEXAS MEDICAL JOURNAL was one of the medical officers embraced in the above list, and having been intimately associated with Medical Director Stout and familiar with much of what is being written, feels a deep interest in the publication. We are very glad indeed that an opportunity has been presented to place this interesting history before the world; it has never been published, and no one but Dr. Stout could publish it. He has all the books and records; and but for the disastrous results of the war, by which he, like all other Confederate soldiers, was impoverished, it would have been published in book form long ago. There has been much talk of publishing it, and certain parties have tried without avail to get possession of the papers. When completed, this work will be a most valuable contribution to the medical history of the war.

---

"Supposin' a Case."—John B. is 20 years of age, and has a spell of sickness. He is thought to be *in extremis* and a priest administer *extreme unction*. The priest is paid a fee. The patient is a step-son of a practicing physician, Dr. C. Patient recovers, and in a short time is of age, and comes into possession of his property, an estate worth $10,000. In his sickness he is faithfully attended by a physician, Dr. A. who is a poor man. The question is, "is John B. entitled to complimentary services from Dr. A?"

The above hypothetical case is sent the JOURNAL for answer with request to submit it to the profession.

Assuming that there are no qualifying conditions which would make the above an exceptional case (such, for instance as perhaps Dr. A. or some member of his family had received or accepted "complimentary services" from, or were under some kind of obligation to the other doctor, the patient's step-father), we should unhesitatingly answer the question in the negative. Indeed, we can scarcely conceive any circumstances under which John B. could *claim* Dr. A.'s services. In the first place, *no one* has a legal or even a moral claim on a physician for gratuitous services. The gratuity must be voluntary. The code says—

"All practitioners of medicine, their wives, and their children, while under the paternal care, are entitled to the gratuitous services of any one or more of the faculty residing near them, whose assistance may be desired." Custom has made it a rule for physicians to not charge each other's family for services, but in any case where the courtesy could not be reciprocated we see nothing to prevent a physician from making a *charge* if he so desire, for services rendered even to a physician's family. And, circumstances alter cases.—The code even stipulates that where a physician in affluent circumstances has the services of a "distant member" of the profession not so well off, the latter "should not refuse an honorarium if offered."

John B. was not a member of Dr. B.'s family; he was not "a child under parental care;" he was a man, and in a few months after his recovery was of legal age, and moreover, was possessed of an independent estate,—i, e., independent of his step-father. Our own opinion is that John B. not only had no right to ask or expect the services of any physician gratuitously but, under the circumstances, he should be ashamed to even think of such a thing, and Dr. A. should not only present his bill, but should make him pay it.

The question, looked at from the standpoint of the code, and of right and justice, will not admit of discussion. It has but one side to it, so far as we can see; but others may not look at it as we do, and we have given it, and our views, for the benefit of those who do not understand the question. If a doctor were obliged to render "complimentary services" to all step-sons who are of age and rich, where is the line to be drawn? He must extend the compliment to this step-son's progeny, even after they come of age; and, so on, *ad infinitum*, as long as he lasts.

However, the question is submitted, and is open for discussion,— the Red Back is not the court of last appeal, nor the oracle. Let us hear the other side, if there be any who differ with us.

---

Surgical Instruments.—A recent number of Meyer Bros. & Co.'s St. Louis *Drug Magazine* states that the Fort Worth Pharmacy Company, of Fort Worth, carries the largest stock of Surgical Instruments and mechanical curative devices in the Southwest. We aré personally acquainted with these people and have a correct knowledge of their stock, fully endorse the above, and will further say, their prices we know to be as low as any of the Eastern houses, and that they are now giving good satisfaction to five hundred Texas doctors whom they number as their patrons. A year ago we asked the profession to aid us in building up this house—a home institution—where orders could be promptly filled and delivered. We have now to say that satisfactory results are being obtained. The Fort Worth Pharmacy Company are also agents for three manufactories of Electric Batteries and two manufactories of Physicians' Chairs. Write to them for what you may want.

